# Genetic Analysis of a Rat Model of Aerobic Capacity and Metabolic Fitness

**DOI:** 10.1371/journal.pone.0077588

**Published:** 2013-10-11

**Authors:** Yu-yu Ren, Katherine A. Overmyer, Nathan R. Qi, Mary K. Treutelaar, Lori Heckenkamp, Molly Kalahar, Lauren G. Koch, Steven L. Britton, Charles F. Burant, Jun Z. Li

**Affiliations:** 1 Department of Human Genetics, University of Michigan, Ann Arbor, Michigan, United States of America; 2 Department of Internal Medicine, University of Michigan, Ann Arbor, Michigan, United States of America; 3 Department of Anesthesiology, University of Michigan, Ann Arbor, Michigan, United States of America; University of Iowa, United States of America

## Abstract

Aerobic capacity is a strong predictor of all-cause mortality and can influence many complex traits. To explore the biological basis underlying this connection, we developed via artificial selection two rat lines that diverge for intrinsic (i.e. inborn) aerobic capacity and differ in risk for complex disease traits. Here we conduct the first in-depth pedigree and molecular genetic analysis of these lines, the high capacity runners (HCR) and low capacity runners (LCR). Our results show that both HCR and LCR lines maintain considerable narrow-sense heritability (h^2^) for the running capacity phenotype over 28 generations (h^2^ = 0.47 ± 0.02 and 0.43 ± 0.02, respectively). To minimize inbreeding, the lines were maintained by rotational mating. Pedigree records predict that the inbreeding coefficient increases at a rate of <1% per generation, ~37-38% slower than expected for random mating. Genome-wide 10K SNP genotype data for generations 5, 14, and 26 demonstrate substantial genomic evolution: between-line differentiation increased progressively, while within-line diversity deceased. Genome-wide average heterozygosity decreased at a rate of <1% per generation, consistent with pedigree-based predictions and confirming the effectiveness of rotational breeding. Linkage disequilibrium index r^2^ decreases to 0.3 at ~3 Mb, suggesting that the resolution for mapping quantitative trait loci (QTL) can be as high as 2-3 cM. To establish a test population for QTL mapping, we conducted an HCR-LCR intercross. Running capacity of the F1 population (n=176) was intermediate of the HCR and LCR parentals (28 pairs); and the F2 population (n=645) showed a wider range of phenotypic distribution. Importantly, heritability in the F0-F2 pedigree remained high (h^2^~0.6). These results suggest that the HCR-LCR lines can serve as a valuable system for studying genomic evolution, and a powerful resource for mapping QTL for a host of characters relevant to human health.

## Introduction

Increased intrinsic exercise capacity or aerobic capacity, generally measured as maximal work with a standardized treadmill test, is an excellent predictor of disease risk in humans, with higher capacities associated with enhanced health and resistance to metabolic disease [1–9]. Peak exercise capacity is a better predictor of mortality than other established risk factors such as hypertension, smoking, and diabetes [7,10]. This frequently observed statistical association suggests a causal connection. However, the biological basis for this connection remains largely unknown [11,12]. Aerobic capacity is a complex phenotype. While some studies document a strong genetic component of maximal oxygen uptake [13,14], other studies report low estimates of heritability [15,16]. In studies involving human subjects, it is often difficult to resolve the relative contributions of genetic and environmental factors to the inter-individual variability of aerobic capacity [13,17–21], e.g., to resolve the relative effects of innate endurance from those due to aerobic training.

To understand the genetic and functional basis of aerobic capacity we sought to establish an animal model that allows in-depth analyses of the biology and health impact of innate aerobic capacity. In 1996, a long-term experiment was initiated to create two lines of rats through bi-directional selection for untrained aerobic running capacity [22]. The two lines, termed high capacity runners (HCR) and low capacity runners (LCR), originated from a founder population of 186 genetically heterogeneous rats derived from outcrossing 8 inbred strains (N:NIH stock) [23]. The animals were selected by their performance in run-to-exhaustion tests on a progressively accelerating treadmill, with the highest and lowest runners, one for each sex from each of 13 families, entering into a rotational breeding scheme (see Methods). 

One of the original aims for establishing the HCR-LCR lines was to test the hypothesis that artificial selection based on intrinsic aerobic capacity would yield models that also exhibit contrasts in disease risks. This hypothesis has been proven correct: after 28 generations of selection, the HCR and LCR diverged not only for running capacity, but also in other physiological measures, including blood pressure, body mass index, lung capacity, lipid and glucose metabolism [24]. The LCR, relative to the HCR, manifest numerous clinically relevant conditions, including increased susceptibility to cardiac ventricular fibrillation [25] and hepatic steatosis [26]. At the behavioral level the LCR score higher for dysfunctional sleep [27], diminished behavioral strategies for coping with stress [28], and impaired memory and learning [29]. In contrast, the HCR have reduced weight gain [30], increased resistance to the deleterious effects of a high fat diet [31,32], increased capacity for fatty acid oxidation in skeletal muscle [33] and liver [26], and a 28-45% increase of lifespan [34].

A major advantage of the HCR-LCR system is that the pedigree and running phenotype data (n = 11,422) are completely known; and tissue samples for most breeding members (n > 1,500) have been archived. This combination of existing data and reagents, combined with over 70 published physiological studies of the two lines, represents a valuable resource that allows comprehensive analyses of the effects of selection on genomic and phenotypic evolution. 

In this study, we carried out a systematic analysis of the running phenotype and related traits over the known pedigree of 0-28 generations. We also collected a genome-wide 10K SNP dataset for a subset of breeding members from three generations (G) (n=142 over G5, G14, and G26), and used these data to examine patterns of genomic evolution in the two lines as they undergo selection. Finally, we performed the first intercross experiments between the HCR and LCR, and analyzed the phenotypic distribution and heritability of the F1 (n = 176) and F2 populations (n = 645). These analyses provided new insights into the genealogical structure, inbreeding patterns, and genetic variability of the two lines, and characterized the intercross animals to assess their suitability as a mapping population for identifying quantitative trait loci (QTL).

## Results

### Rotational breeding and inbreeding coefficients

**Figure 1:**
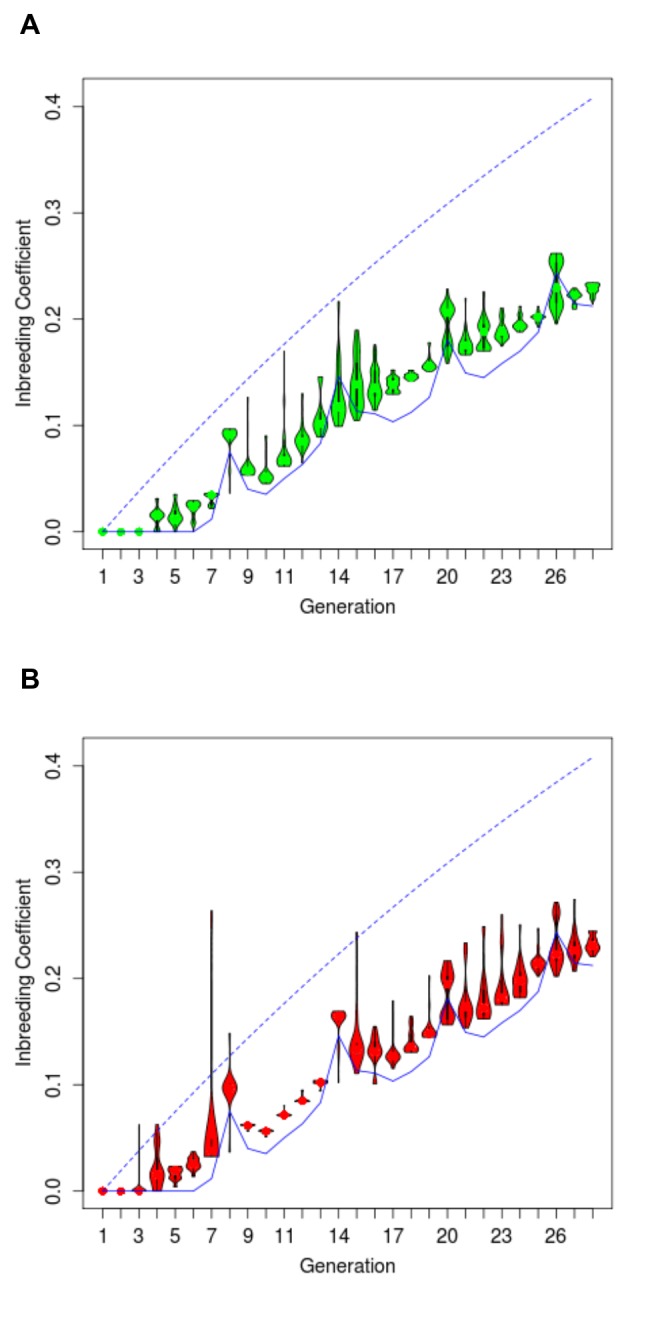
Distribution of predicted inbreeding coefficients (F) for generations 0 to 28. Shown are "violin-plots" for individual generations for HCR (**A**) and LCR (**B**). The widths of the ovals indicate the probability density of the data values. The black dots and the thick black lines in the ovals denote the median and the 25-75 percentile range, respectively. The dotted blue line indicate the expected increase in F under random mating given the 13-family breeding scheme, and the solid blue lines indicate the expected F under perfect adherence to the rotational breeding scheme.

The protocols of animal maintenance, phenotyping, and rotational breeding have been described previously [22] (see also Methods). We analyzed the pedigree data for generations 1-28 (Files S1-S2), involving 5,976 HCRs and 5,446 LCRs. For each animal, we calculated its expected inbreeding coefficient (F) by tracing its parental lineage and documenting inbreeding loops. As expected, such pedigree-based estimates of F started to rise at G4-G5 and continued to increase over successive generations (Figure **1**). The breeding history included occasional out-of-schedule pairings due to the lack of offspring of a certain sex in a given family or the need to substitute for unproductive mating pairs (see Methods for more details). Despite this, the pattern of F increase in actual pedigrees largely agrees with the expectation assuming perfect adherence to the planned rotation schedule (shown in solid lines in Figure **1**). The cyclic rise of inbreeding coefficient every six generations is expected for 13 breeding pairs, due to the inevitable first-cousin paring every half cycle of the rotation [35,36] (further explained in Methods). Importantly, the average increase of estimated F is 0.94% and 0.95% per generation for HCR and LCR, respectively. These predictions are slower than the rate expected under random mating (shown in dotted lines in Figure **1**), which increase at 1.51 per generation for both HCR and LCR, starting from the first generation. 

### Phenotypic response to selection and heritability

**Figure 2:**
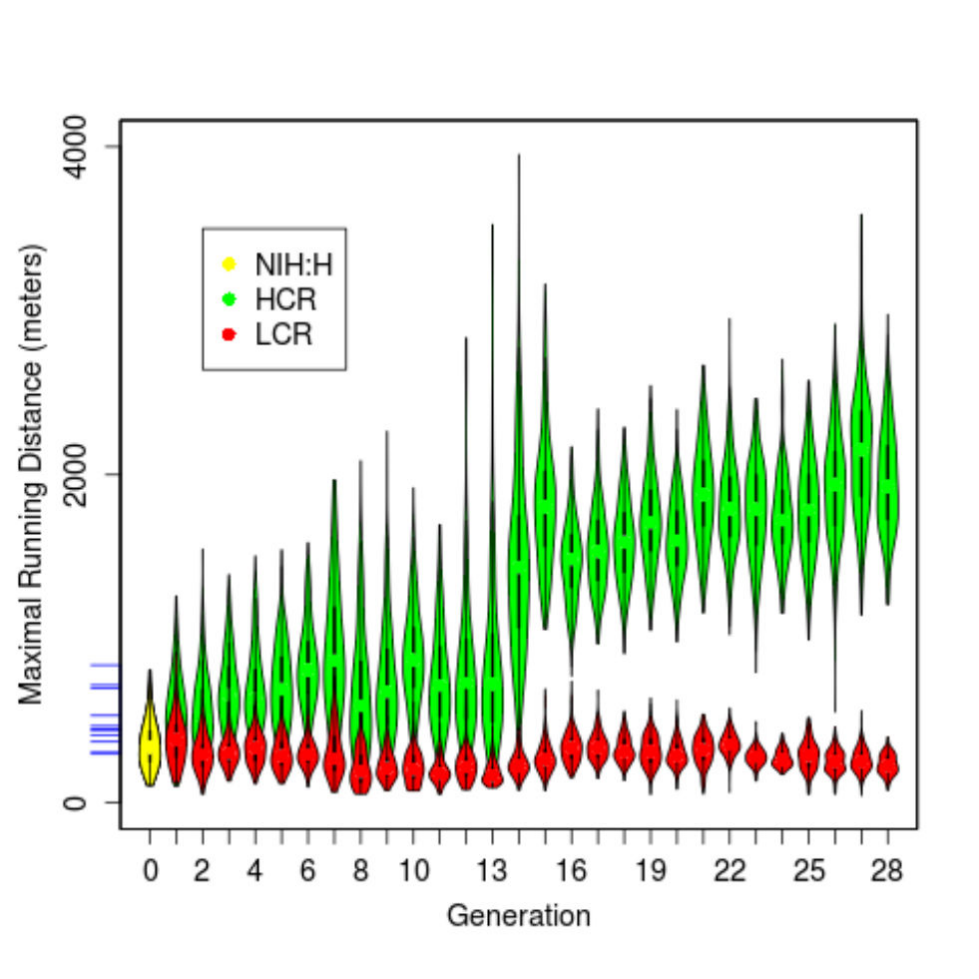
Distribution of maximal running distance for generations 0 to 28. Shown are "violin-plots" for individual generations for females and males combined. The yellow oval to the left denotes the founder population (NIH:H, n=153 phenotyped, out of 186), while green and red ovals are for HCR and LCR, respectively. The blue tick marks on the y axis indicate the maximal running distance for eleven inbred lines, which are ordered, from top to bottom, as DA (840m), PVG (718m), AUG (699m), SR (533m), F344 (469m), ACI (450m), LEW (442m), WKY (414m), BUF (373m), MNS (308m) and COP (298m).

**Figure 3:**
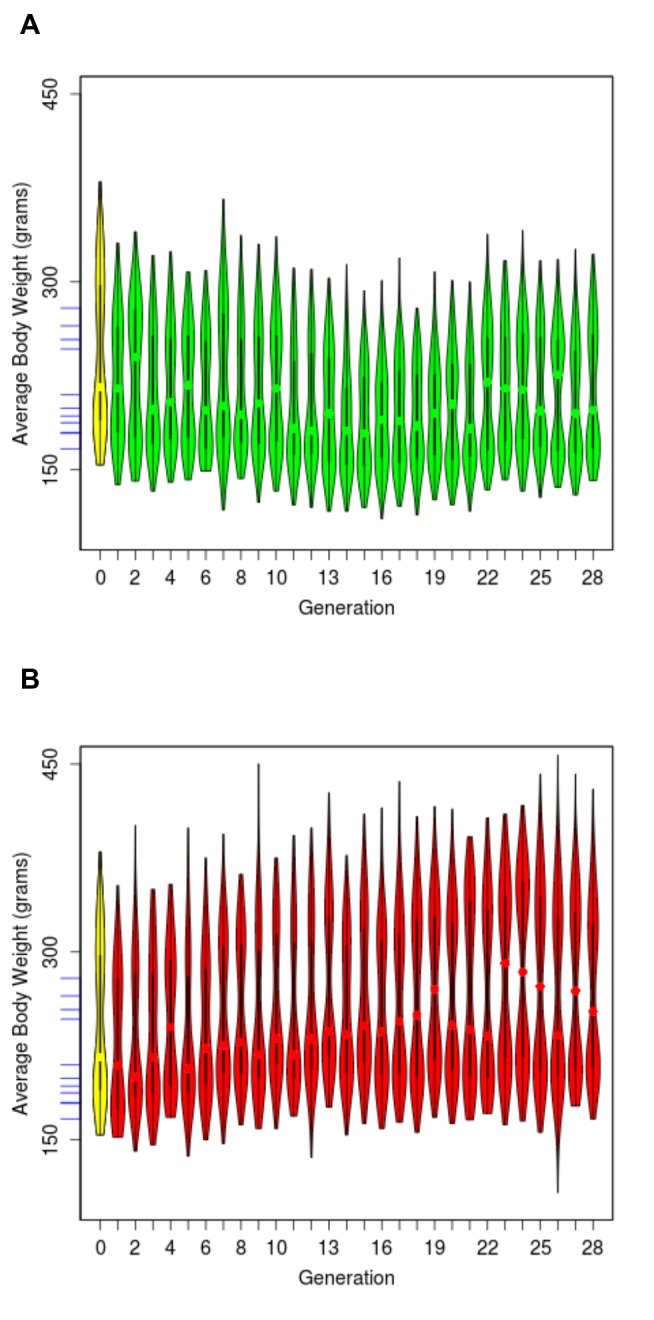
Distribution of body weight for generations 0 to 28 for HCRs (**A**) **and** **LCRs** (**B**). The yellow oval denotes the founder population (NIH:H, n=153). Most distributions are bi-modal, as most females are of lower body weight than most males. The blue tick marks on the y axis indicate the body weight for eleven inbred lines. They are ordered, from top to bottom, as MNS (279g), LEW (265g), SR (254g), BUF (246g), WKY (210g), COP (199g), ACI (193g), F344 (188g), DA (180g), PVG (179g) and AUG (167g).

For each animal, we collected phenotype data that include maximal running distance, body weight at the time of running trial, and vertical work performed during each run. All animals were tested at 11-12 weeks of age. While both lines were derived from the same base population (indicated in yellow in Figure **2**), their running performance gradually diverged over time. After 28 generations, the HCRs and LCRs differ by about 8.3-fold in running distance (9 times of the average within-line standard deviation), compared to ~2.8 fold (range of 298 to 840 meters) among eleven inbred lines commonly used in research [37]. The HCR continue to respond to selection (Figure **2**, Table **1**-**2**), with maximal running distance reaching >2000 m, ~2.4 fold higher than the best recorded performance among the inbred lines [37]. The pattern of increase is consistent in both males and females (Figure **S1**). Body weight increased in LCR and decreased in HCR In the first 12-13 generations, but did not diverge further after G13, stabilizing to a 0.7 to 0.8-fold difference through 28 generations (Figure **3**, Table **1**-**2**). In general, females are of lighter weight than males. However, as females tend to run longer, the overall vertical work is near-equivalent between males and females (1.3-fold difference in HCR, 1.1-fold difference in LCR) and larger in HCR than LCR by 6.8 fold.

**Table 1:** Summary of cohort size, running distance, and body weight by gender and by generation for HCRs.

	Male					Female				
		Best Running Distance (meters)		Body Weight (grams)			Best Running Distance (meters)		Body Weight (grams)	
Gen	n^†^	Mean	SD	Mean	SD	n^†^	Mean	SD	Mean	SD
1	57	529.0	244.3	265.7	27.5	59	564.7	224.3	181.2	20.7
2	82	495.3	201.9	271.3	29.0	55	652.2	263.0	174.2	16.7
3	74	630.4	215.1	258.2	29.3	86	721.9	248.5	175.0	18.3
4	64	611.3	190.2	261.4	25.4	67	784.3	270.4	176.0	15.9
5	86	661.6	218.0	252.9	26.2	65	812.9	270.0	171.9	14.2
6	79	769.9	220.0	250.6	28.2	72	916.5	271.1	169.7	13.4
7	67	722.6	305.7	274.5	34.5	66	1116.2	420.6	174.4	17.9
8	96	543.7	313.0	263.9	35.4	89	795.8	420.8	174.1	15.7
9	118	600.0	272.8	257.4	29.3	116	889.2	381.9	172.3	17.0
10	140	799.0	261.6	257.7	32.6	98	1018.7	308.0	172.3	17.5
11	92	641.2	281.3	246.2	30.5	110	849.3	314.0	164.5	17.6
12	90	756.1	348.6	251.3	27.1	105	883.5	528.5	166.4	16.0
13	134	729.4	386.5	236.8	28.6	106	1024.2	651.6	158.8	19.1
14	112	1243.2	443.2	220.9	32.2	110	1703.6	602.7	154.9	16.2
15	122	1667.0	284.5	221.0	28.5	110	2023.8	424.5	152.1	15.1
16	106	1476.0	253.0	224.1	25.1	104	1475.0	236.9	158.0	17.2
17	146	1509.2	248.9	227.4	28.1	137	1595.6	264.7	157.4	17.8
18	149	1540.9	241.1	220.8	29.4	121	1658.5	256.4	156.5	17.5
19	126	1669.0	271.8	225.7	26.2	101	1787.4	263.0	160.1	15.4
20	135	1579.2	246.0	233.7	26.0	113	1661.3	233.2	156.6	15.0
21	115	1834.0	306.7	233.1	27.1	120	1934.1	291.0	160.2	14.6
22	140	1729.6	250.1	254.2	28.2	118	1902.0	279.5	167.8	16.0
23	122	1697.3	279.0	254.0	31.0	105	1867.1	290.6	173.5	16.8
24	110	1649.4	226.5	258.0	25.7	105	1834.2	264.9	173.8	18.5
25	124	1657.0	279.2	257.7	25.2	140	1913.6	258.5	172.5	15.2
26	168	1834.9	277.7	251.8	23.1	129	2053.1	357.0	163.5	12.1
27	145	2000.7	364.3	246.3	26.3	145	2295.2	392.6	163.8	14.8
28	112	1810.4	269.6	261.4	26.1	113	2108.6	297.5	169.1	14.0

† Total number of animals generated, including animals produced for line maintenance and resource sharing.

**Table 2:** Summary of cohort size, running distance, and body weight by gender and by generation for LCRs.

	Male					Female				
		Best Running Distance (meters)		Body Weight (grams)			Best Running Distance (meters)		Body Weight (grams)	
Gen	n^†^	Mean	SD	Mean	SD	n^†^	Mean	SD	Mean	SD
1	61	372.3	157.1	280.6	29.0	64	421.1	176.7	178.3	17.2
2	58	268.7	116.4	296.7	31.2	68	333.8	123.6	187.5	12.9
3	86	309.5	119.5	287.3	34.2	81	325.3	96.2	186.3	17.2
4	72	302.6	89.2	297.6	27.8	66	366.9	106.0	194.7	16.0
5	58	252.4	73.0	299.9	38.4	92	326.1	104.9	194.7	19.1
6	74	281.7	73.0	293.8	31.1	81	333.9	108.8	195.7	19.2
7	78	263.0	139.2	312.7	29.5	63	356.3	150.3	203.8	17.6
8	70	157.7	93.4	313.6	24.2	73	259.6	104.8	209.4	23.9
9	95	171.7	65.3	320.8	39.3	103	260.3	81.8	205.5	20.6
10	76	167.3	94.4	321.9	25.6	74	245.0	88.5	213.8	18.8
11	124	156.5	60.9	318.6	38.7	121	217.7	72.4	207.2	20.8
12	98	182.8	90.6	321.4	36.5	104	267.9	102.8	211.9	21.5
13	109	150.4	51.6	336.2	31.5	107	215.2	69.5	216.9	16.7
14	98	205.8	57.8	304.2	32.6	111	266.4	78.4	207.2	20.9
15	111	240.3	75.9	318.3	40.4	120	318.7	102.7	214.4	21.4
16	63	303.2	69.3	314.3	29.9	78	370.0	107.3	208.0	28.0
17	127	318.3	75.6	316.8	35.5	127	367.4	87.2	203.8	19.4
18	127	282.6	58.2	323.5	32.8	119	374.6	69.3	202.7	18.0
19	123	281.3	78.4	327.7	32.1	115	363.9	92.0	213.6	19.1
20	113	272.3	64.0	326.7	29.2	120	330.7	87.3	208.0	19.4
21	112	291.3	90.4	336.3	30.7	114	356.3	83.8	203.9	19.2
22	99	328.9	63.6	335.1	30.8	105	389.2	65.8	202.3	16.6
23	103	248.3	38.0	339.8	32.3	88	322.5	45.7	210.8	17.6
24	88	238.1	30.1	354.9	24.3	84	308.9	47.0	214.8	19.2
25	115	228.0	65.2	340.6	33.2	110	346.5	73.3	205.3	17.7
26	117	201.7	45.1	327.0	50.3	123	305.3	49.4	206.2	19.4
27	135	217.8	49.6	331.6	31.7	119	309.3	64.1	206.4	14.4
28	115	191.8	42.9	328.2	31.5	111	278.3	45.8	208.6	16.7

† Total number of animals generated, including animals produced for line maintenance and resource sharing.

The narrow-sense heritability (h^2^) for the logarithm of running distance, which measures the proportion of total phenotypic variance explained by additive effects of genes, was calculated for each line separately, and was 0.47 ± 0.02 in HCRs and 0.43 ± 0.03 in LCRs when all 28 generations were considered. To evaluate potential change in heritability over time, we also calculated h^2^ over four-generation, partially overlapping, intervals and found that while h^2^ was variable across intervals, it maintained positive values, with no sign of abatement in later generations (Figure **S2**). The within-line h^2^ for bodyweight and vertical work are 0.45 ± 0.06 and 0.37 ± 0.02, respectively, for HCRs, and 0.17 ± 0.03 and 0.58 ± 0.02 for LCRs. These results indicate that although LCRs did not show a decrease of running capacity as dramatically as the increase in HCRs, the heritability of running performance was comparable in the two lines. Lower body weight is associated with better running capacity, as shown by the negative correlations between the two phenotypes for both sexes within each line. For HCRs, the spearman correlation is -0.41 ± 0.15 and -0.17 ± 0.16 for males and females, respectively. For LCRs, the correlation is -0.19 ± 0.16 and -0.12 ± 0.13 for males and females, respectively. The fact that HCRs continued to respond to selection and that both lines maintained within-line heritability suggest that causal genetic variants have not been fixed in either line, rather they continue to segregate in both pedigrees. 

### Increased genomic differentiation between lines

**Figure 4:**
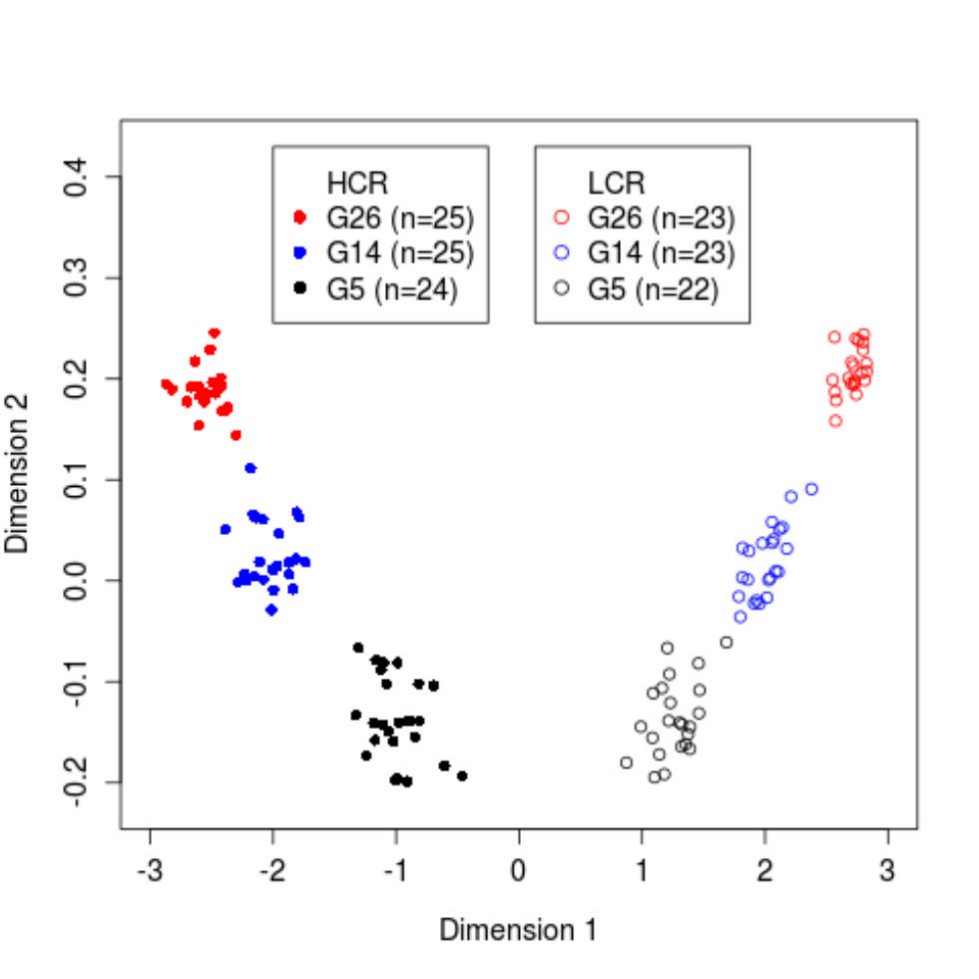
Progressive genetic differentiation revealed by 10K SNP genotyping data. Shown is a multidimensional scaling plot (dimensions 1 vs. 2) for 142 genotyped animals in two lines and three generations, as indicated by different symbols, showing that the two lines formed separate clusters at G5, and diverged further in G14 and G26.

As an initial genetic characterization of the HCR-LCR system, we collected genotype data over a genome-wide panel of ~10K single nucleotide polymorphism (SNP) loci for 142 animals, consisting of 22-25 animals in each of three non-adjacent generations (G5, G14, and G26) in both lines (see Methods). We chose these three generations to profile the long-term genomic changes in the two lines.. We used the average heterozygosity of 61 X chromosome (ChrX) markers to infer sex (Figure **S3A**), and found no disagreement with the recorded sex of the 142 animals. Documented relatedness was also confirmed by plotting the pairwise proportion of not sharing DNA segments due to identity by descent (P(IBD) = 0, or Z0) against the proportion of sharing one copy IBD (Z1) using the 2,518 SNPs (SNP Panel-2, see Methods). Siblings and non-sibling relatives are separated into distinct clusters (Figure **S3B**), indicating that the sample identities reflected in the genotype data are consistent with the recorded pedigree. Multidimensional Scaling (MDS) analysis showed that at G5, HCRs and LCRs formed two readily separable clusters (Figure **4**). From G5 to G14 and from G14 to G26, between-lines separation increased, indicating a progressively greater differentiation between LCR and HCR. 

To measure the apportionment of total genetic variance into between-line and within-line components we performed an Analysis of Molecular Variance (AMOVA) [38]. The proportion of variance explained by among population difference, as a weighted average over all loci, is increasing over time, from 6.5% at G5, to 15.6% at G14, and to 26.5% at G26. 

### Decreased genomic diversity within lines

**Figure 5:**
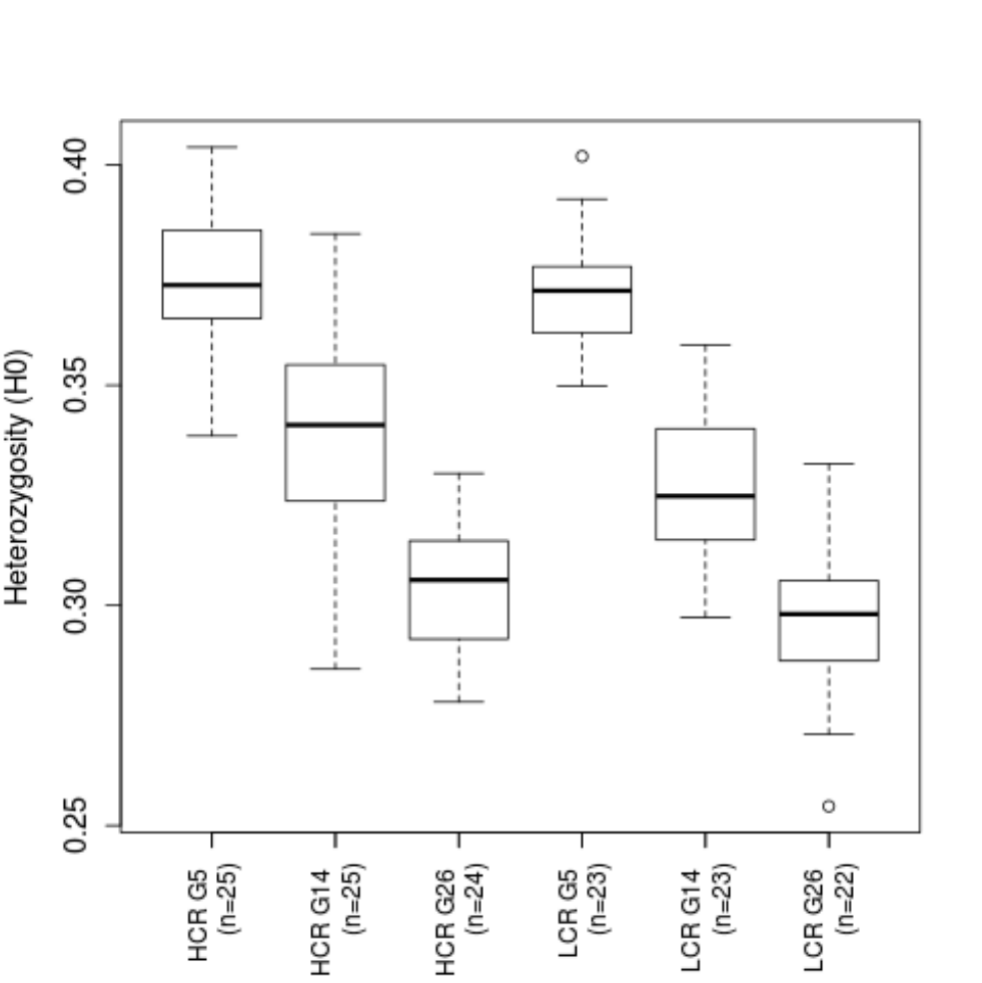
Decrease of average heterozygosity over time in both lines. Shown is the boxplot of genomewide average heterozygosity for the genotyped animals in two lines and three non-adjacent generations.

We analyzed genetic diversity at the individual level by calculating the average heterozygosity (H_0_) across the 2,518 Panel-2 markers for each animal, and averaging within each of the six groups (two lines, at three time points) (Figure **5**). At G5, H_0_ averaged 0.379 in HCR and 0.372 in LCR. At G14 it decreased to 0.338 (-10.8%) in HCR and 0.327 (-12.1%) in LCR. At G26 it decreased further to 0.303 (-10.2% from G14) in HCR and to 0.296 (-9.4%) in LCR. Note that the absolute values of H_0_ are influenced by the allele frequencies of the genotyped SNP markers, and it is the relative changes of H_0_ that reflect the altered genomic diversity. The observed rates of decrease are slower than in random mating populations. Using the known numbers of effective breeders at each generation we calculated the expected H_0_ at each generation assuming random mating, and found that the expected rate of decrease in H_0_ is on average 1.53 per generation for both HCR and LCR,. The observed rate of decrease, 0.95% and 0.97% per generation for HCR and LCR, respectively, is lower by 37-38%, consistent with the pedigree-based predictions (Figure **1**) and confirming that the rotational breeding scheme has successfully reduced inbreeding as predicted [39]. 

As the reduction of H_0_ over time primarily reflects higher levels of inbreeding, a majority of the increase of homozygote genotypes should be accounted for by the emergence or expansion of long runs of homozygosity (ROH). Using 10,185 SNPs in SNP Panel-1 (see Methods), we found that for HCR, ROH covered an average of 46% of the genome in G5 animals, and this rate increased to 54.8% in G26. For LCR, ROH covered 46.8% of the genome in G5, and 55% in G26. Thus the non-ROH regions shrink by 0.73-0.77% per generation in the two lines, accounting for most of the decrease of H_0_.

### Linkage disequilibrium

**Figure 6:**
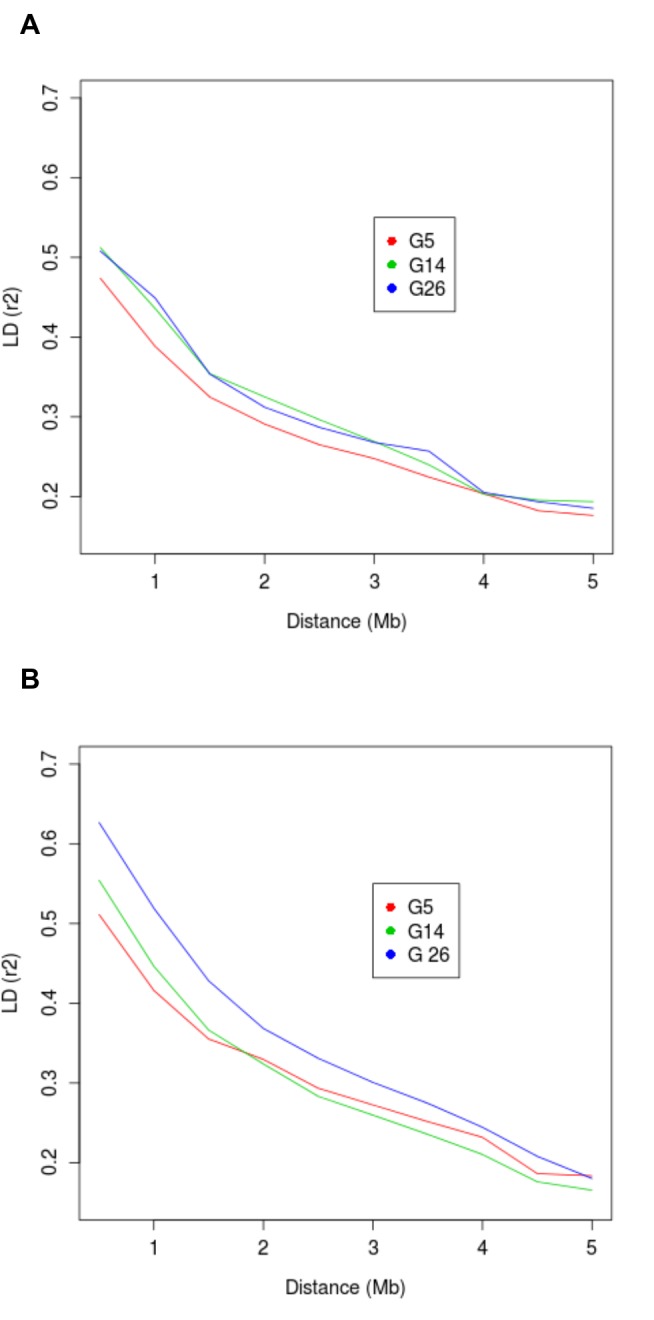
Linkage disequilibrium (LD) decay over distance in HCR (A) and LCR (B) for chromosome 1. The LD index r^2^, averaged for marker pairs falling in discrete distance bins, were plotted against the distance in Mb. LD decays to r=0.3 in about 3 Mb for both HCRs and LCRs.

We examined linkage disequilibrium (LD) patterns using the Panel-1 SNPs on Chromosome 1 (n = 978). The LD index r^2^ decays to 0.3 at ~3 Mb in both HCR and LCR (Figure **6**). The level of LD is similar between HCR and LCR, showing slightly higher r^2^ in later generations, and is consistent with those reported for the NIH Heterogeneous Stock [40]. These results also suggest that the resolution of QTL mapping using HCR-LCR can be as high as 2-3 cM, considerably higher than the 20-40 cM resolution of F2 intercross of inbred lines [41].

### "F2" intercross of HCR-LCR

As HCR and LCR have evolved separately, a direct between-line comparison of phenotypes and genotypes would incur the effect of population stratification. To create a QTL mapping population with randomized genomes, we performed "F2" intercross experiments using 28 HCR-LCR pairs and obtained 242 F1 animals (176 phenotyped). From the phenotyped F1 population we set up 63 mating pairs and produced 645 F2 animals. The term "F1" or “F2” is applied loosely in this context because our crosses were not based on inbred lines. However, we use F0, F1, and F2 to indicate the generations. 

**Figure 7:**
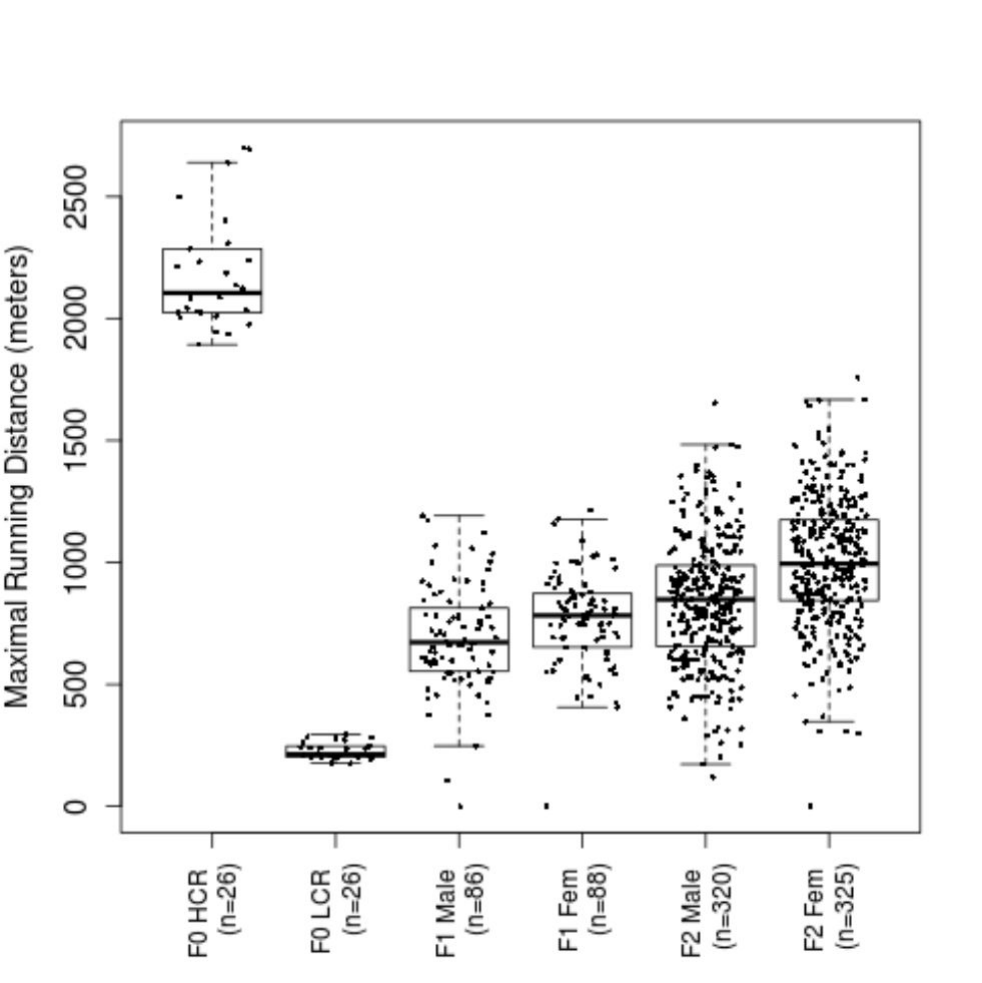
Distribution of running performance for animals of the F2 intercross experiment. Shown are boxplots of the best running distance by generation and by sex.

The running phenotype of F1 fell in an intermediate range between that of their HCR and LCR parents, and the F2 animals exhibited larger variations than the F1 rats (Figure **7**). This pattern is consistent with the model in which most neutral alleles no longer co-segregate with the phenotypes; and at functional loci (i.e., those responsible for the phenotypic differences between HCRs and LCRs), F1s tend to be heterozygous and F2s carry a wider assortment of genotypes.

Importantly, the h^2^ of the maximal running distance in the F0-F2 pedigree remained high, at 0.60 ± 0.05. The h^2^ for vertical work is comparable to those of the ancestral lines under selection (0.61 ± 0.05), while the h^2^ for body weight is lower (0.03 ± 0.04). In addition, we phenotyped a number of other physiological measures in a subset of F2 animals, including heart/body mass ratio (n=380), extensor digitorum longus (EDL) mass/body mass ratio (n=387), and percent body fat (n=490), which showed strong heritability (0.42 ± 0.11, 0.36 ± 0.10, and 0.48 ± 0.10, respectively). These results indicate that the running capacity and related traits are influenced by genetic factors, confirming that it is feasible to use the F2s as a mapping population for QTL.

## Discussion

While previous studies have focused on functional, physiological comparisons between HCR and LCR rats, here we conducted the first in-depth pedigree and molecular genetic analysis of the two lines. Phenotypic data over the 28-generation pedigree not only revealed substantial heritability for the running capacity trait, but also showed that the heritability is maintained in later generations, suggesting continued selection. In addition, the strong heritability is recapitulated in the F2 intercross. These findings suggest that causal variants continue to segregate in both HCRs and LCRs and persist in the F2 population. Our study generates the first direct evidence that the trait under selection is highly heritable, providing justification for intercross-based QTL mapping. In addition, the heritability for vertical work, heart/body ratio, EDL/body ratio, and percent body fat suggests that the model can be used for simultaneous QTL mapping for multiple traits.

We observed continued response to selection in HCR during 20-28 generations. This observation is notable because it can be interpreted in two possible scenarios. The first is that the aerobic running capacity may be influenced by many interacting QTLs, and as variants in some loci become fixed under selection, the previously hidden phenotypic effects of other variants can be "released" and come under selection, thereby fueling prolonged responsiveness. This agrees with previous observations in similar systems that long-term selection did not exhaust the genetic variation for the selected trait due to the graduate shifts in the capacitors of cryptic genetic variation [42,43]. The second scenario is that the trait may be governed by many QTL of small effects, hence the strength of selection (~20% of animals become breeders in each generation (see Table **3**)) may not have effectively driven the rapid changes of allele frequencies. The two scenarios are not mutually exclusive, and the observation means that not all causal alleles have been differentially fixed in the two lines. Therefore the F2 mapping approach needs to consider the possibility that the causal variants may be segregating *within* one or both lines. 

**Table 3:** Number of phenotyped animals (N) by line and generation, and the number of animals chosen as effective breeders (N_b_), separately shown for male (N_m_) and female (N_f_).

			HCR				LCR	
Gen	N^†^	N_b_	No. Male (N_m_)	No. Female (N_f_)	N^†^	N_b_	No. Male (N_m_)	No. Female (N_f_)
0	-	26	13	13	-	26	13	13
1	116	28	14	14	125	26	13	13
2	137	28	14	14	126	26	13	13
3	160	28	14	14	167	30	15	15
4	131	26	13	13	138	26	13	13
5	151	28	14	14	150	26	13	13
6	151	30	15	15	155	26	13	13
7	133	30	15	15	141	26	13	13
8	185	28	13	15	143	28	14	14
9	234	30	14	16	198	27	14	13
10	238	29	13	16	150	27	13	14
11	202	34	16	18	245	28	14	14
12	195	30	14	16	202	26	13	13
13	240	34	16	18	216	28	14	14
14	222	30	15	15	209	28	14	14
15	232	32	16	16	231	30	15	15
16	210	38	19	19	141	30	15	15
17	283	42	21	21	254	38	19	19
18	270	46	23	23	246	38	19	19
19	227	42	20	22	238	45	22	23
20	248	44	22	22	233	50	25	25
21	235	50	25	25	226	39	19	20
22	258	48	24	24	204	50	25	25
23	227	48	24	24	191	52	26	26
24	215	44	22	22	172	50	25	25
25	264	50	25	25	225	44	22	22
26	297	44	22	22	240	50	25	25
27	290	-	-	-	254	-	-	-
28	225	-	-	-	226	-	-	-
Total	5976	967	476	491	5446	920	459	461

† All the offspring generated including backup lines.

HCRs exhibited accelerated improvement of running capacity during G12-G15 (Figure **2**). To identify the cause(s) of this acceleration we examined factors such as diet, running protocols, the breeding schedule, and "Operator", i.e., the experimenter or a team of experimenters assessing the running phenotype. The average litter size (i.e., fecundity) in the recorded HCR pedigree was not changed significantly during this period (Figure **S4A**), hence there was no noticeable change in fertility or the strength of selection (i.e., the fraction of animals chosen as breeders). There was no systematic correlation between litter size and inbreeding coefficient of the offspring (not shown); and there was no documented change in diet, running apparatus, or running protocol. The breeding schedules for the two lines were closely synchronized across all 28 generations (Figure **S5A**). The pedigree-based prediction of F was increasing in both HCR and LCR as expected (Figure **1**). However, a more detailed retrospective analysis of the breeding records found three factors having changes during the G12-G15 period. The first is Operator: a team supervised by Operator 3 performed the running tests during G7-G13, while a team supervised by Operator 4 performed the tests during G14-G15. The second factor is the number of animals in the pedigree with no entries for running data which accrued mostly from rats that "refused" to run. The number of non-compliant rats in both lines gradually increased during G7-G13 in both lines, dropped immediately at G14, and remained low for most of subsequent generations (Figure **S4B**). Despite presumed standardization of the running protocol, the loss of running data may be Operator dependent in the sense that "refusal to run" is a subjective measure. Third, the fraction of mating pairs that were out-of-schedule increased in G12-G14 in HCR, and dropped after G15 (Figure **S4C**). The simplest interpretation of these co-occurrences is that Operator 3 subjectively determined that a large number of animals refused to run. Those who did run showed no improvement over G6-G13. With Operator 4, nearly all animals were able to run, and ran better than previous generations. While plausible, this simple scenario does not explain all the observations. First, despite being kept and tested under the same conditions as the HCRs, the LCRs exhibited no comparable acceleration or deceleration in running capacity. Second, the acceleration in HCRs began in G12-G13 with the unexplained emergence in some families of one or two exceptional runners, whose running distance were often more than twice as long as that of their siblings (Figure **S6**). The performance of these runners could not be easily explained by Operator. Partly because the exceptional runners tended to be selected as breeders, such improved performance spread wider across the cohort in G14-G15 and gradually became the norm after G16. However, there was not a clear-cut Mendelian segregation pattern in these generations: the pairing of two exceptional runners often still produced mediocre offspring. Among HCR mating pairs in G12-G15 there were 13 out-of-schedule pairs, which did not produce more exceptional runners than on-schedule pairs (not shown). The location of animal facility changed between G15 and G16 for both lines, but this change took place after the acceleration had started. Despite these complications, heritability estimates for HCR, when calculated for three-generation sections of the pedigree and shifted by one generation, did not show dramatic changes over the generations (Figure **S5B**). 

The accelerated improvement of running capacity in HCRs during G12-G15 could also reflect genetic changes. However, emergence of a single high-impact *de novo* mutation is unlikely, as prodigious running capacity arose in multiple families concurrently. Such a pattern, however, is compatible with a scenario in which causal "high" alleles in multiple genes interact in a non-linear fashion. Various combinations of the high alleles could have undergone gradual enrichment and in G12-G15, began to manifest as improved phenotype when the most favored combinations were formed. Future studies, including linkage analysis of these intermediate generations, are needed to characterize the genetic changes accompanying the apparent varying tempo of trait evolution. 

In the F2 generation of the intercross, the running distance distribution is wider than in F1, but did not reach the full range seen in F0 animals. The fact that none of the F2 animals performed as well as their HCR grandparents, and very few performed as poorly as the LCR grandparents, strongly suggests that multiple genetic loci are involved. The Castle-Wright estimator of the effective number of QTLs is calculated as 4-10 using our F2 data [44,45]. Caution should be taken as the calculation is based on simplifying assumptions such as unlinked loci of equal effects that have no interaction. Only the actual linkage or association studies can reveal the number and impact of QTL underlying the trait in question. 

The HCR-LCR system was initiated in 1996 [22] and reached G28 in 2011. During this time, the two lines have diverged in innate endurance running capacity and showed marked differences in body type and metabolic traits. The HCR animals show a lower weight gain than LCR, in both young and adult rat, and this can be partly accounted for by higher spontaneous activity and lower fuel economy during activity in HCRs [32]. The two lines also diverged for many health indicators, with HCR showing a relative resistance to obesity, higher insulin sensitivity, lower blood pressure, improved lipid parameters, and enhanced longevity [30,31,34]. These phenotypes are of immense public health interest, as prevalence of diabetes, cardiovascular disorders, obesity, and metabolic syndrome is rising at an alarming rate and account for a major portion of disease burden worldwide [46]. The model system used in this study is ideally suited for elucidating the fundamental biology of metabolic health. Understanding the genetic architecture and molecular underpinnings of the remarkable HCR-LCR differences has the potential to provide new insights into the relationship between exercise capacity and metabolic health in humans. 

Taken as a whole, the results presented above suggest that the HCR-LCR system is well-suited to serve as a novel model system for studying genome evolution under sustained selection and for dissecting the functional and genetic basis of polygenic traits. The model exhibits large phenotypic divergence, sustained heritability for a wide range of cardiovascular and metabolic traits, and maintained outbred character. The complete pedigree is known, with running phenotype for all animals already collected, and tissue sample for most breeders archived. Compared to inbred line-based gene mapping, our system offers some additional advantages. First, while the F2 generation could be subjected to conventional linkage mapping [47], the two lines have accumulated ~60 generations of historical recombination (~30 as the NIH Heterogeneous Stock, 28 generations of divergent selection, and F2 intercross). Consequently, animals in both lines carry fine-grained genomic mosaics of eight "ancestral" inbred strains, with LD structure on the order of 3 Mb, allowing for greater resolution in association analysis [48–50]. Second, our system has maintained genetic diversity through rotational breeding, such that networks of interacting QTLs may have evolved jointly under selection, making the system particularly suitable for detection of interaction QTL [51]. Combining QTL mapping with the wealth of existing knowledge of the HCR-LCR system is expected to allow the identification and prioritization of high quality candidate genes that will shed insight into the biology of oxidative capacity and metabolic fitness.

## Materials and Methods

### Ethics statement

This study was approved by the University Committee on Use and Care of Animals, Ann Arbor, Michigan (Approval Numbers: #08905 and #03797). The proposed animal use procedures are in compliance with University guidelines, and State and Federal regulations. 

### Rotational breeding scheme

In practice, each line contains at least 13 mating pairs through all generations. From each of the family produced, one male and one female are selected as breeders for the subsequent generation. For HCR, the male and female with the greatest running distance are selected, whereas in LCR, those with the lowest distance are selected. The breeders are paired between different families to avoid brother-sister mating, and the pairings rotate in successive generations to minimize inbreeding [39]. When the 13th rotation is reached, same-family mating is skipped, and the pairings are reiterated starting again in the same way as rotation 1 (Figure **S7A**). Sometimes, if a particular mating fails, or if a family lacks animal of one sex, substitute mating is attempted involving a male from another family (Table 2). In some cases, one male is mated to two females. After G12, female HCR with extremely low body weights were not selected to be breeders in order to avoid reduced fecundity [52]. During G9-G13 in HCR there were also three cross-generation matings, whose offspring were incorporated into subsequent generations. Further, occasionally additional pairs are bred to generate experimental cohorts for study by us or for sharing with collaborators, and the progenies in these "analytical families" are not used for maintaining the lines, and are not counted in our calculation of the expected inbreeding levels (see below). They are, however, used to calculate heritability and the distribution of trait values. 

One inevitable consequence of this breeding scheme is the mating of first-cousins at every half interval (Figure **S7B**). For example, at G7, every breeding pair, such as 1M-7F (a male from Family 1 and a female from Family 7) involves first cousins, because they are from 1F-7M and 7F-13M matings respectively, in G6, in which 7M and 7F are siblings. This results in a 6% spike in inbreeding values in G8 (Figure **1**), and such a cyclic pattern continues in subsequent generations, resulting in spikes at G14, G20, and G26. The actual pedigree deviates from a perfectly executed breeding scheme due to the inclusion of substitute breeders, and the resulting estimates of inbreeding coefficient from the actual pedigree depart slightly from the expectations (Figure **1**). As some breeding pairs were assembled to generate offspring for research use rather than line propagation, the "effective breeders" are those that contribute offspring who are also used as breeders, and do not include those whose offspring were used only for research (Table **3**). 

### Running phenotype

Eleven week old animals are subjected to run-to-exhaustion tests without prior training, except for brief sessions of treadmill education during the week prior to the tests. The purpose of such education sessions is to familiarize the rats to the experimenters and the testing equipment and to ensure that each rat has the ability to achieve a minimal level of continual running for 5 minutes at least once, which constitutes the threshold performance necessary for inclusion in the actual running tests the following week. During education, the rats learn to keep running in order to avoid a mild shock (1.2 mA of current at 3 Hz) induced by the electrified grid located at the back of the treadmill. For all sessions the treadmill is set at a 15-degree upward slope.

During the run-to-exhaustion test, each rat was evaluated on five consecutive days (Mon-Fri) for G0-G16 and on three alternating days (Mon-Wed-Fri) for G17-28. Each trial starts at a velocity of 10 m/min, which increases by 1 m/min every 2 min until the rat reaches exhaustion. Exhaustion point is defined as the third time a rat can no longer keep pace with the treadmill and remains on the shock grid for two seconds rather than resuming running. At this point the rat is removed from the treadmill and weighed. For each rat, the best distance out of the multiple trials is taken as the best estimate of its intrinsic capacity, and used as the criterion for breeder selection. The vertical work during each trial is estimated using the equation:

*work* = (*running distance*) x (*body weight*) x (*sin[15°*]) x (*9.8m/s* ^*2*^)/1000 in which the unit for work is joule (J=kg•m^2^/s^2^). Unit for running distance and body weight is meter and gram, respectively.

### Phenotype distribution

To display phenotype distribution we produced violin plots (as shown in Figures **1**, **2**, and **3**) using the *vioplot* function from the *Vioplot* package in R. To calculate the Spearman's rank correlation coefficient (ρ) between maximal running distance and body weight, separately for two sexes within each line, we used the cor and cor.test functions in R. The ρ values were calculated for each generation, and averaged over G1-G28.

### Inbreeding coefficient and Heritability

We calculated the inbreeding coefficient (F) for each animal in the pedigree using the *calcInbreeding* function from the *pedigree* package in R [53]. The pedigree for earlier generations of NIH:H animals, i.e., those that preceded the founders of our lines, was not available. We are therefore limited to calculate the *increase* of inbreeding coefficients from those of the founders, effectively assuming they were unrelated, while in fact they were related according to the (unknown) breeding patterns in the preceding generations. To calculate the expected F under random mating, we used the equation F_n+1_ = F_n_+(N_f_+N_m_)/(8*N_f_*N_m_)-F_n_(N_f_+N_m_)/(8*N_f_*N_m_), where the F_n_ and F_n+1_ are the inbreeding coefficients at the n-th and (n+1)-th generation, respectively, and N_f_ and N_m_ are the numbers of male and female breeders at the n-th generation (N_f_ = N_m_ = 13 in every generation for a 13-family breeding scheme). To calculate the expected F under perfect adherence to the rotational scheme, we generated an idealized pedigree of 13 mating pairs of exact mating patterns as intended, and used the *pedigree* package to calculate F for every member of the pedigree.

To calculate the narrow-sense heritability (h^2^), we applied the variance and covariance component models as implemented in *SOLAR* version 4.3.1 [54]. We estimated h^2^ for maximal running distance both over the entire pedigree and for four-generation intervals (with one and three generation overlap) to assess the h^2^ variation over time. We also estimated h^2^ for body weight and work for the G0-G28 pedigree, and for additional traits for the F2 intercross. For the G0-G28 analysis, we included sex and operator as covariates; and for the F2 intercross we included sex and batch because the breeding was performed in two batches, containing 154 and 491 F2 animals, respectively.

### Genotyping and data processing

DNA from 22-25 breeders from both lines in three non-adjacent generations (G5, G14, and G26, n=142) was extracted from frozen liver tissue, and genotyped across 10,846 SNP loci using the Affymetrix Rat Mapping 10K GeneChip. Attempts to extract DNA from generations earlier than G5 revealed that many samples in G0 and G4 were degraded. We therefore chose G5 as the earliest generation in our analysis due to its assured DNA quality. In assessing the quality of SNP markers we removed 28 duplicate SNPs, 496 SNPs with genotype missing rate >10%, and 137 SNPs with Hardy-Weinberg Equilibrium test p < 0.001. These steps led to 10,185 SNPs that formed the "Panel-1" markers. As some analyses require a reduced set of SNPs without rare variants and without strong linkage disequilibrium, we removed from Panel-1 an addition set of 7,284 SNPs selected by trimming SNP pairs in linkage disequilibrium with r^2^ value >0.05 (in windows of 10 SNPs, sliding by 2 SNPs each time), and 572 SNPs with minor allele frequency (MAF) <5%. After these steps, 2,518 SNPs remained and formed the "Panel-2" markers. The Panel-2 markers were used in calculations of IBD, AMOVA, and genome-wide average heterozygosity. Pairwise Identity-by-State (IBS) matrix was estimated in *PLINK* [55] using the *-genome* command and Panel-2 markers. Multidimensional scaling analysis of the IBS matrix was performed in R [56]. Analysis of molecular variance (AMOVA), as implemented in the program *Arlequin*, was used to calculate the within- and among-group differentiation [57]. 

We assessed the accuracy of recorded sex for each genotyped animal by calculating the average heterozygosity of the X chromosome SNPs. Male and female animals are confirmed by non-overlapping distributions of ChrX heterozygosity values. We confirmed known sibling pairs among the genotyped animals by plotting pairwise Z0 vs Z1 values in R. Z0 and Z1 values were determined in *PLINK* using the *-genome* command.

### Runs of homozygosity

Using the 10,185 Panel-1 markers, we identified long runs of homozygosity (ROH), in *PLINK* using the -*homozyg* command. We defined ROHs as genomic segments with at least 4 homozygous markers and having a density of at least 1 SNP per 500Kb. Total ROH length in each animal was obtained by summing over all ROH and also reported as the fraction of the rat genome (2.75 Gb).

### Genomewide average heterozygosity

Using the Panel-2 markers, we calculated the average heterozygosity in PLINK using the *-hardy* command, and compared across genotyped lines and generations using boxplots. The expected heterozygosity values were calculated using the equation H_n+1_ = H_n_(1-((N_f_+N_m_)/(8*N_f_*N_m_))), where the H_n_ and H_n+1_ are the heterozygosity at the n-th and (n+1)-th generation, respectively, and N_f_ and N_m_ are the numbers of male and female breeders at the n-th generation, respectively (N_f_ = N_m_ = 13 in every generation for a 13-family breeding scheme) (Table **3**). 

### LD calculation

Pairwise measurements of LD (r^2^) were calculated for marker pairs within 5 Mb on Chromosome 1 using Haploview [58]. Chromosome 1 was chosen as a representaive autosome. To show the relationship between r^2^ and inter-marker distance, we calculated average r^2^ values for groups of marker pairs falling in discrete bins of inter-marker distance, in 500Kb increments, and plotted the values for G5, G14, and G26 in both HCR and LCR (as shown in Figure **6**). 

### “F2” intercross

We performed the F2 intercross in two batches. For the first batch, we randomly selected 4 males and 4 females from G26 of each line to form 8 HCR-LCR reciprocal pairs, which generated 79 F1 rats, from which 20 males and 20 females were randomly selected to form pairs between different F1 families (i.e., avoiding brother-sister mating). This generated 154 F2 rats. For the second batch, we selected 9 males and 9 females from G28 of each line to form 18 mating pairs, which generated 163 F1 rats, from which 97 were phenotyped. Out of the 97 F1 animals, 40 males and 40 females were selected across the 18 families with equal representation between HCR/LCR parentage, such that we had 4 combinations (HCR-mom male with HCR-mom female, HCR-mom male with LCR-mom female, LCR-mom male with HCR-mom female, and LCR-mom male with LCR-mom female) and generated 491 F2 rats. The two batches together yielded 645 F2 rats (Figure **7**). Phenotyping for running performance followed the same protocols as described above. Additionally, for F2 animals in the second batch we measured lean mass, fat mass, fluid mass, fasting blood glucose, heart mass, and EDL muscle mass at 16-20 weeks of age. Body composition was determined via NMR using Bunter Optics Minispec LF90 II. Blood glucose after a 4-hour fast was determined using Accu-Check Aviva meter. At time of dissection, heart and EDL muscles were weighed immediately upon harvesting.

## Supporting Information

Figure S1 **Distribution of maximal running distance for generations 0 to 28 for males (**A**) and females** (**B**). Shown are "violin-plots" for individual generations for females and males separately. The blue tick marks on the y axis indicate the maximal running distance for eleven inbred lines, which are ordered, from top to bottom for males (**A**) as DA (968m), PVG (754m), SR (615m), AUG (594m), ACI (447m), LEW (405m), WKY (387m), BUF (355m), F344 (332m), MNS (302m) and COP (262m), and for females (**B**) as AUG (805m), DA (712m), PVG (682m), F344 (606m), LEW (479m), ACI (453m), WKY (441m), SR (409m), BUF (391m), COP (333m), and MNS (315m).
(TIFF)

Figure S2 **Narrow-sense heritability of running capacity remained positive over time.** Shown are heritability (h^2^) estimates and standard errors for maximal running distance in four-generation intervals that overlap by one-generation for HCR (**A**) and LCR (**B**).
(TIFF)

Figure S3 **Sample quality assessment in 10K SNP genotype data.** (**A**) Average heterozygosity over 61 SNPs on the X chromosome for 142 genotyped animals, ordered by line and generation. Males and females fall in two non-overlapping clusters, indicating that the observed X-chromosome heterozygosity is consistent with the reported sex of the animals. (**B**) Scatter plot of Z0 (genomic proportion that a pair of animals share 0 allele identical-by-descent [IBD]) versus Z1 (proportion that a pair of animals share 1 allele IBD), showing that known sib pairs form a separate cluster than more distant relatives.
(TIFF)

Figure S4 **Assessment of litter size, missing phenotype, and rotational breeding schedule over the generations.** (**A**) Average litter size over G1-G28 for the HCR animals in the recorded pedigree, accompanied by standard deviations among all the families for each generation. No significant change in litter size is observed over the course of selection. (**B**) Number of animals in each generation without recorded running phenotype for HCR (blue) and LCR (red). The increase in missing phenotypes between G7-G13 overlaps operator 3 (shown as the orange bar below). (**C**) Percent of mating pairs out-of-schedule (off-rotation) per generation for HCR (green) and LCR (red).
(TIFF)

Figure S5 **Between-line synchrony and short-term heritability estimates in HCR.** (**A**) Dates of birth (x-axis) for G1-G28 animals (y-axis). The horizontal bars indicate the range of birth dates, and the dots indicate the average. The close match between HCR and LCR shows that the two lines are synchronized. (**B**) HCR narrow-sense heritability for adjacent 3-generation intervals, showing no significant change over time.
(TIFF)

Figure S6 **Running performance of mid-parents and offspring for on/off-rotation mating types for G12 (**A**), G13 (**B**), G14 (**C**), and G15 (**D**).** Shown are the scatterplots of mid-parent running distance (x-axis) versus male and female offspring running distance (y-axis) for on-rotation mating (indicated by XX.0), off-rotation mating due to mother (XX.1), off-rotation mating due to father (XX.2), off-rotation mating due to both mother and father (XX.3), and either father or mother from the enrichment cohort (XX.4). Data points above the solid diagonal line indicate offspring with better running performance than their mid-parent.
(TIFF)

Figure S7 **Rotational breeding scheme.** (**A**) Mate pairing matrix for breeding rotations 1 through 12, where females from families 1 through 13 are designated in columns (from left to right), while males are designated in rows (from top to bottom). Male-female pairs are formed differently in successive generations as indicated by the rotation numbers in the matrix. (**B**) An example to show how, at the mid-cycle through rotational breeding among 13 families, every breeding pair is a first-cousin mating. The example shown is rotation 7 between Family 1 and Family 7. A male from family 1 (1M) to be mated with a female from family 7 (7F) are both offspring of breeding members of family 7 in rotation 6, thus making them first cousins.
(TIFF)

File S1 **HCR pedigree in G1-G28 and select phenotypes.**
(XLSX)

File S2 **LCR pedigree in G1-G28 and select phenotypes.**
(XLSX)
