# Correction: Genetic Analysis of a Rat Model of Aerobic Capacity and Metabolic Fitness

**DOI:** 10.1371/journal.pone.0092345

**Published:** 2014-03-24

**Authors:** The PLOS ONE Staff

There are multiple errors in Supplementary Information File S2. A corrected version of File S2 can be viewed here:

## Supporting Information

File S2 **LCR pedigree in G1-G28 and select phenotypes.**
(XLSX)
