# Supplementary figures and images for: Genetic Analysis of a Rat Model of Aerobic Capacity and Metabolic Fitness
Source: PLoS One. 2013 Oct 11;8(10):e77588. doi: 10.1371/journal.pone.0077588 (PMC3795692; doi:10.1371/journal.pone.0077588)

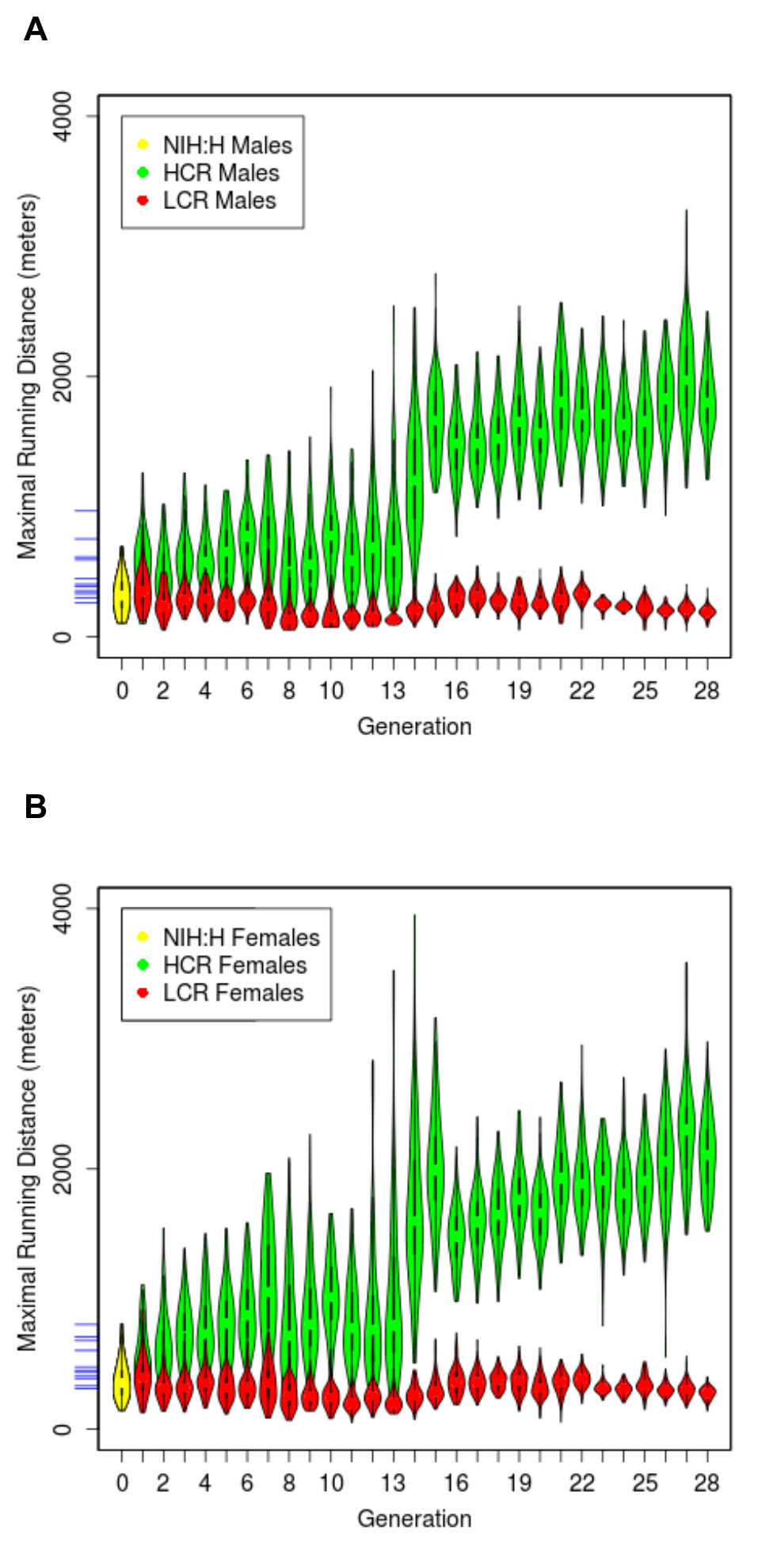

Supplement: Figure S1 — Distribution of maximal running distance for generations 0 to 28 for males (A) and females (B). Shown are "violin-plots" for individual generations for females and males separately. The blue tick marks on the y axis indicate the maximal running distance for eleven inbred lines, which are ordered, from top to bottom for males (A) as DA (968m), PVG (754m), SR (615m), AUG (594m), ACI (447m), LEW (405m), WKY (387m), BUF (355m), F344 (332m), MNS (302m) and COP (262m), and for females (B) as AUG (805m), DA (712m), PVG (682m), F344 (606m), LEW (479m), ACI (453m), WKY (441m), SR (409m), BUF (391m), COP (333m), and MNS (315m). (TIFF) [file pone.0077588.s001.tiff]

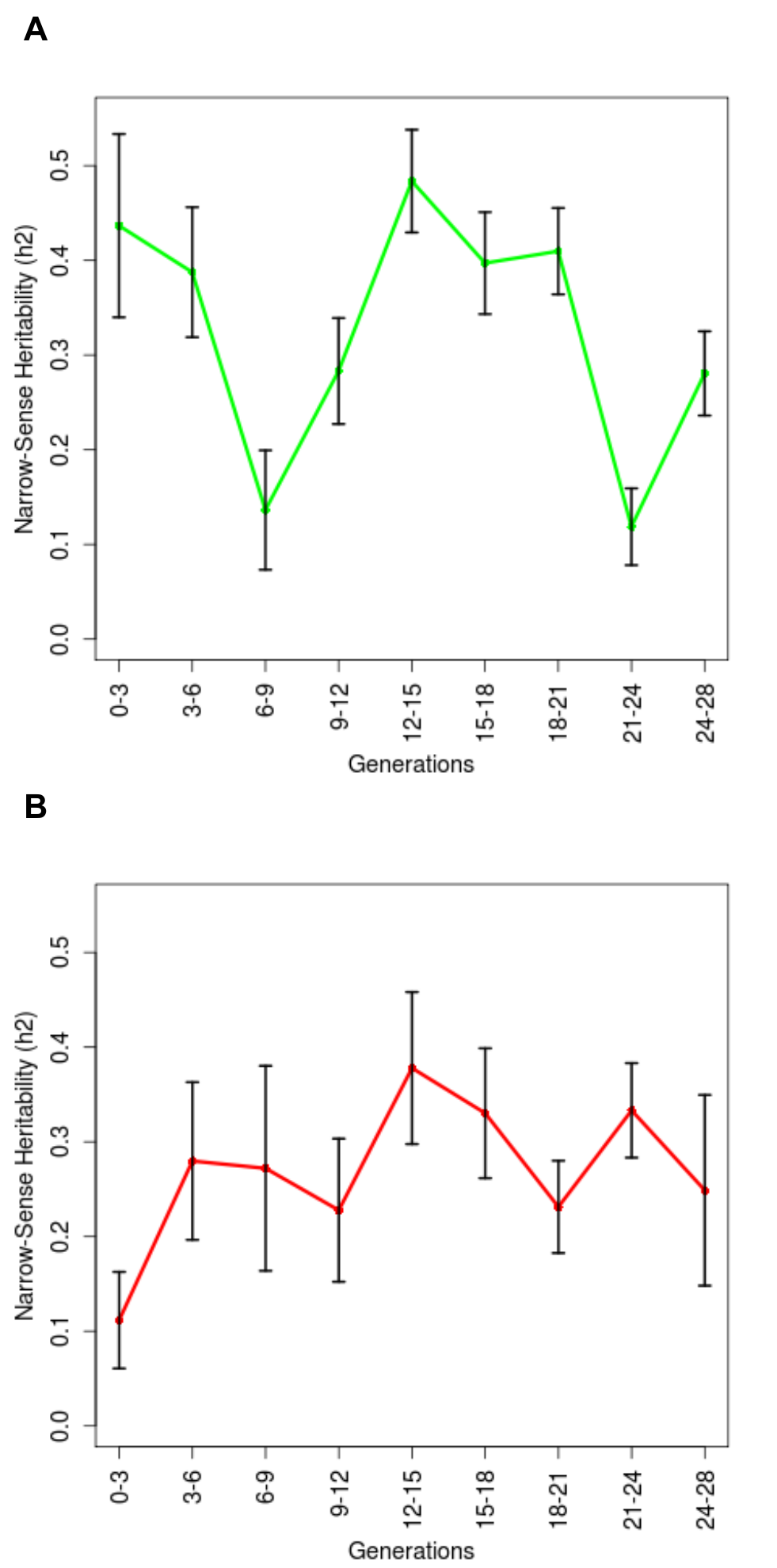

Supplement: Figure S2 — Narrow-sense heritability of running capacity remained positive over time. Shown are heritability (h2) estimates and standard errors for maximal running distance in four-generation intervals that overlap by one-generation for HCR (A) and LCR (B). (TIFF) [file pone.0077588.s002.tiff]

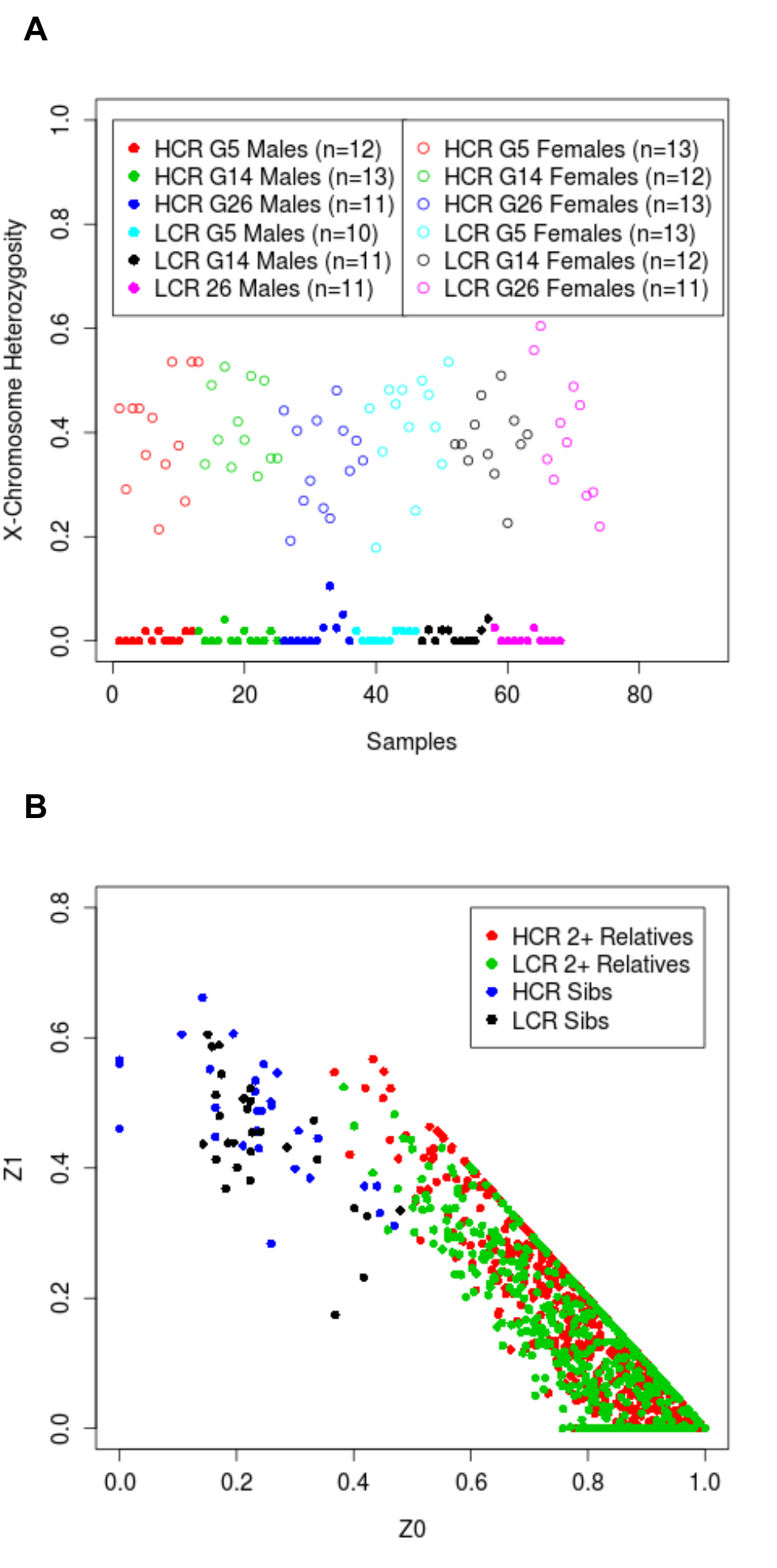

Supplement: Figure S3 — Sample quality assessment in 10K SNP genotype data. (A) Average heterozygosity over 61 SNPs on the X chromosome for 142 genotyped animals, ordered by line and generation. Males and females fall in two non-overlapping clusters, indicating that the observed X-chromosome heterozygosity is consistent with the reported sex of the animals. (B) Scatter plot of Z0 (genomic proportion that a pair of animals share 0 allele identical-by-descent [IBD]) versus Z1 (proportion that a pair of animals share 1 allele IBD), showing that known sib pairs form a separate cluster than more distant relatives. (TIFF) [file pone.0077588.s003.tiff]

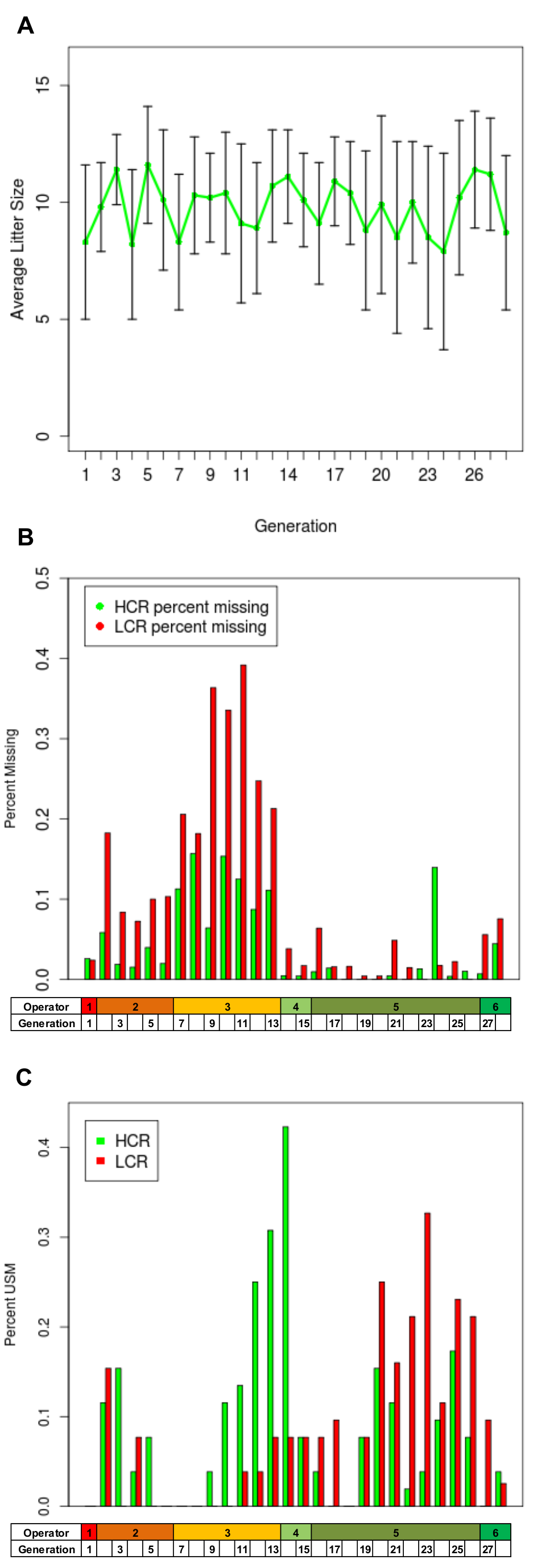

Supplement: Figure S4 — Assessment of litter size, missing phenotype, and rotational breeding schedule over the generations. (A) Average litter size over G1-G28 for the HCR animals in the recorded pedigree, accompanied by standard deviations among all the families for each generation. No significant change in litter size is observed over the course of selection. (B) Number of animals in each generation without recorded running phenotype for HCR (blue) and LCR (red). The increase in missing phenotypes between G7-G13 overlaps operator 3 (shown as the orange bar below). (C) Percent of mating pairs out-of-schedule (off-rotation) per generation for HCR (green) and LCR (red). (TIFF) [file pone.0077588.s004.tiff]

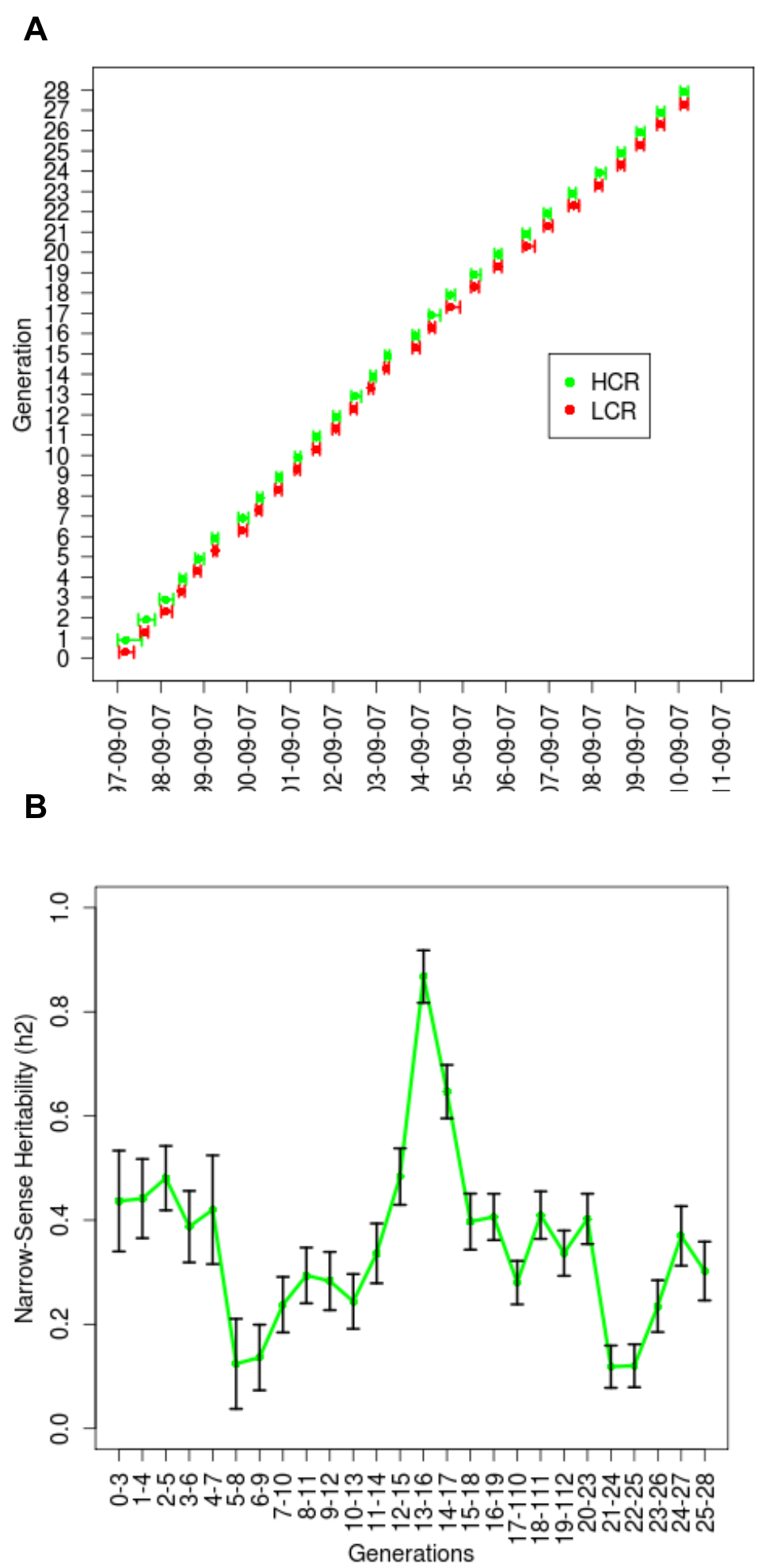

Supplement: Figure S5 — Between-line synchrony and short-term heritability estimates in HCR. (A) Dates of birth (x-axis) for G1-G28 animals (y-axis). The horizontal bars indicate the range of birth dates, and the dots indicate the average. The close match between HCR and LCR shows that the two lines are synchronized. (B) HCR narrow-sense heritability for adjacent 3-generation intervals, showing no significant change over time. (TIFF) [file pone.0077588.s005.tiff]

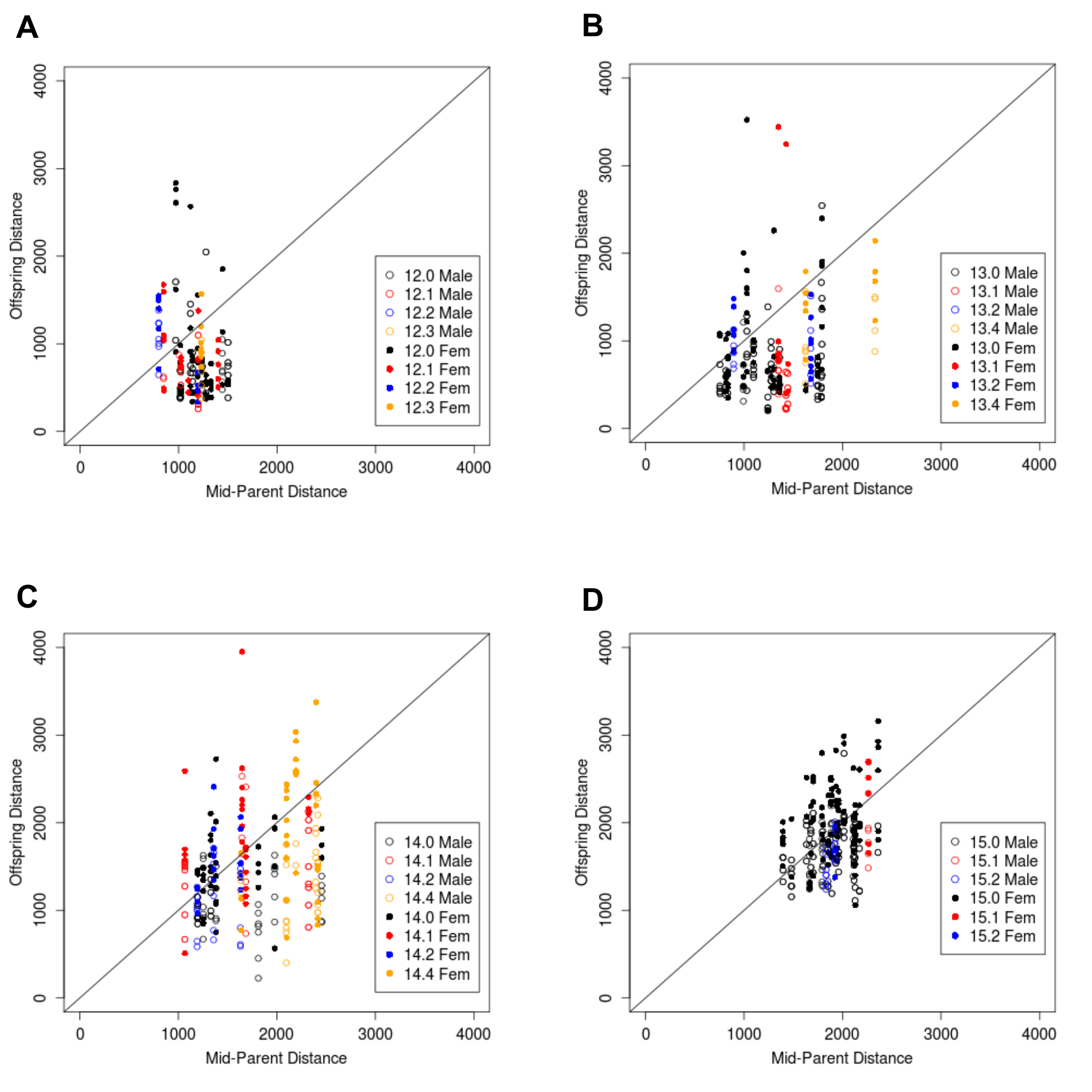

Supplement: Figure S6 — Running performance of mid-parents and offspring for on/off-rotation mating types for G12 (A), G13 (B), G14 (C), and G15 (D). Shown are the scatterplots of mid-parent running distance (x-axis) versus male and female offspring running distance (y-axis) for on-rotation mating (indicated by XX.0), off-rotation mating due to mother (XX.1), off-rotation mating due to father (XX.2), off-rotation mating due to both mother and father (XX.3), and either father or mother from the enrichment cohort (XX.4). Data points above the solid diagonal line indicate offspring with better running performance than their mid-parent. (TIFF) [file pone.0077588.s006.tiff]

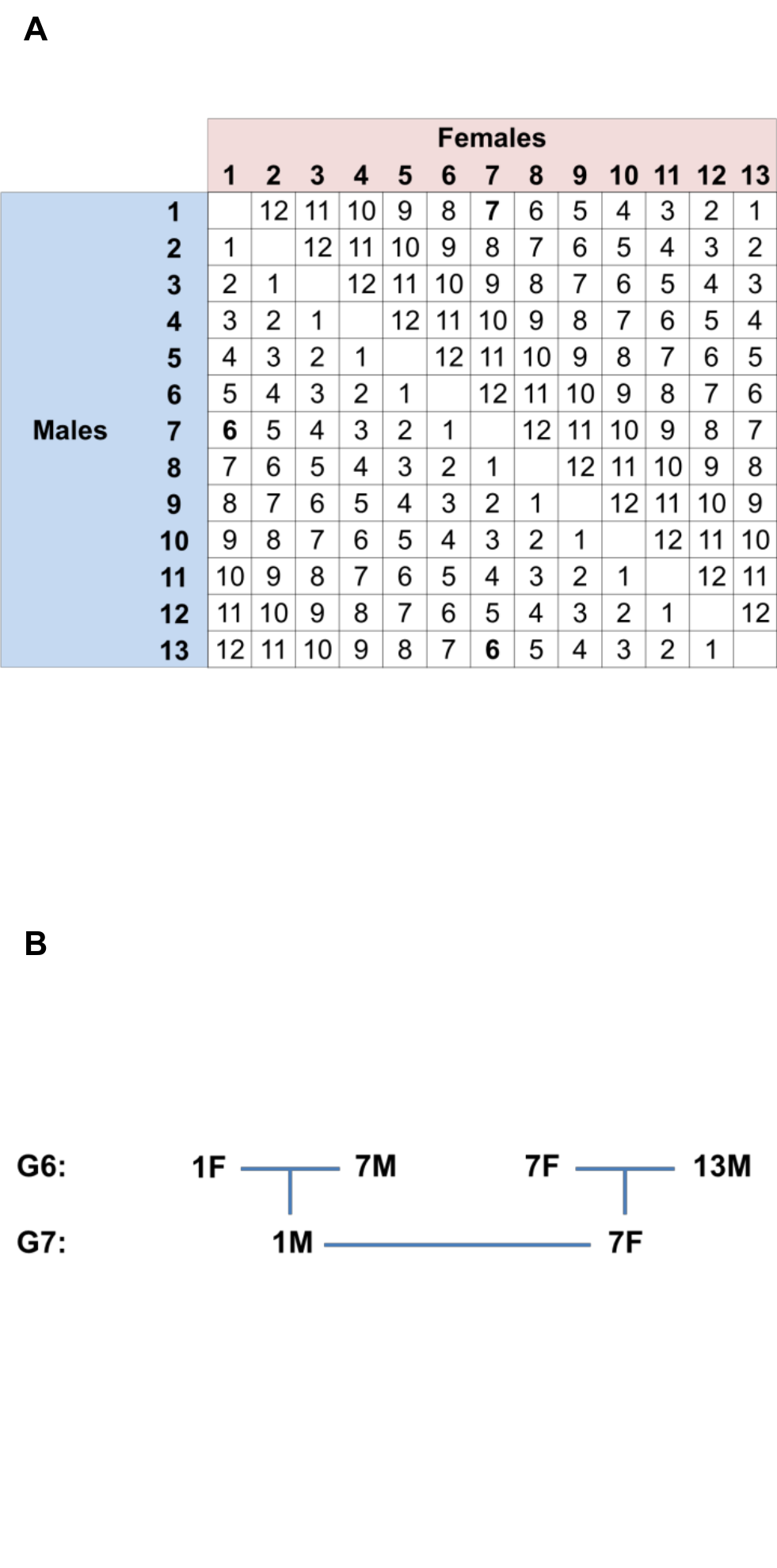

Supplement: Figure S7 — Rotational breeding scheme. (A) Mate pairing matrix for breeding rotations 1 through 12, where females from families 1 through 13 are designated in columns (from left to right), while males are designated in rows (from top to bottom). Male-female pairs are formed differently in successive generations as indicated by the rotation numbers in the matrix. (B) An example to show how, at the mid-cycle through rotational breeding among 13 families, every breeding pair is a first-cousin mating. The example shown is rotation 7 between Family 1 and Family 7. A male from family 1 (1M) to be mated with a female from family 7 (7F) are both offspring of breeding members of family 7 in rotation 6, thus making them first cousins. (TIFF) [file pone.0077588.s007.tiff]
